# Efficacy of Core Muscle Exercise Combined with Interferential Therapy in Alleviating Chronic Low Back Pain in High-Performance Fighter Pilots: A Randomized Controlled Trial

**DOI:** 10.1186/s12889-024-18177-7

**Published:** 2024-03-05

**Authors:** Chongwen Zuo, Zhiyang Zheng, Xiaoyan Ma, Fen Wei, Yushui Wang, Yi Yin, Shuai Liu, Xiaosong Cui, Chaoqun Ye

**Affiliations:** 1 Department of Rehabilitation Medicine, Air Force Medicine Centre of Chinese PLA, 100142 Beijing, China; 2 Beijing Sports University, 100091 Beijing, China; 3 Tianjin University, 300072 Tianjin, China

**Keywords:** Core muscle exercise, Interferential current, Physical therapy, Chronic low back pain

## Abstract

**Background:**

Chronic low back pain (LBP) related to flight is a prevalent health issue in military aviation, impacting pilots. The objective of this investigation was to ascertain if the application of core muscle training in conjunction with interferential current (IFC) therapy results in a reduction in pain severity and associated disability, consequently enhancing core muscle functionality in Chinese Air Force high-performance fighter pilots experiencing chronic LBP.

**Methods:**

Fifty-three fighter pilots with chronic LBP were randomized into 3 groups: a core muscle exercise combined with IFC group (CG, *n* = 19), a core muscle exercise group (EG, *n* = 19), and an IFC group (IG, *n* = 15). The three groups underwent therapeutic intervention 5 times a week for 12 weeks. The primary outcomes were pain intensity, Oswestry Disability Index (ODI) score and SF-12 health-related quality of life (PCS and MCS) score. Secondary outcomes included evaluations of trunk muscle strength, endurance, and range of motion (ROM) during medial/lateral rotation to assess muscle functionality. Measurements were obtained both before and after the implementation of the intervention therapy.

**Results:**

After 12 weeks of intervention therapy, all the health condition parameters significantly improved among the three groups. However, the CG had a significant improvement in pain intensity compared to the EG (MD = − 0.84 scores; 95% CI = − 1.54 to − 0.15; *p* = 0.013) and the IG (MD = − 1.22 scores; 95% CI = − 1.96 to − 0.48; *p* = 0.000). Additionally, the CG led to greater conservation of ODI and improved SF-12 PCS scores than did the IG (*p* < 0.05). Finally, compared with those at baseline, the core muscle function parameters in the CG and EG improved significantly at the end of the study, but no statistically significant differences were observed between the two groups (*p* > 0.05).

**Conclusion:**

Among participants with chronic LBP, three intervention therapies appear effective in reducing pain, diminishing disability, and enhancing quality of life. Also, combined therapy significantly improved pain and disability compared to the other two monotherapies; moreover, combined therapy and core muscle exercise provided similar benefits in terms of core muscle function after 12 weeks of intervention therapy.

## Introduction

Chronic non-specific low back pain (LBP) has become increasingly prevalent among high-performance fighter pilots due to the continually rising intensity of their flight-related training experiences [1, 2]. LBP has been reported by one out of every three fighter pilots [2], and nearly 10–50% of fighter pilots have reported radiological evidence of lumbar disc degeneration [3]. The higher incidence of LBP in fighter pilots may be associated with increased exposure to elevated flight stressors, including alterations in body posture, nonergonomic practices, high acceleration loads, and the use of head equipment. Additionally, the ability of fighter pilots to endure the strain of prolonged confined sitting and the number of hours flown during high accelerating forces has led to significant shock, which indicates that high stability is required by the spine during flight training, while pilots with lower strength and trunk muscle resistance are at high risk of LBP [4]. These noncombat-related injuries have become the leading cause of troop attrition in modern warfare; therefore, an effective strategy should be used to decrease the prevalence of LBP in fighter pilots.

The sitting position itself is not associated with LBP, but prolonged sedentary and restricted sitting positions increase the risk of LBP [5]. It has been reported that intradiscal pressure is increased in the prolonged sitting posture, and cause a negative effect on the nutrition of the intervertebral disc [6]. Although the static posture is bearable, prolonged periods of exercise and changes in sitting angles are necessary to provide periodic relief and optimal physiological status of muscles [7]. Flight-related LBP can, in turn, be induced by prolonged periods of static posture and muscular fatigue [8, 9]. Generally, pain and fatigue can be relieved by adjusting the sitting position; however, opportunities for this change are restricted in high-performance aircraft seats due to limited space and body-mounted safety gear.

It has been reported that with the development of anti-G clothing, fighter pilots do not seem to perform anti-G maneuvers (maximum contraction of the transverse abdomen muscle) as often as they used to; thus, these pilots promote a reduction in the tone of the core stabilizing muscle group [10]. There is indisputable evidence that these risk factors contribute to an excessive spinal load, leading to muscle fatigue and tension, ultimately culminating in alterations in spinal structure [11, 12]. Grossman et al. [2] identified that the average intensity of back pain among fighter pilots was classified as mild to moderate. While this degree of pain falls within the limits of tolerance and is not considered severe, it has raised significant concerns among various military pilots. If effective measures to prevent and treat pain are not taken, it will directly threaten pilots’ development, such as lost workdays, an aviator’s health check-up, and a loss of in-flight performance, these adverse consequences will pose great challenges to the career development of pilots and national defense security [13].

Among the conservative approaches for the management of chronic non-specific LBP, physical therapy and therapeutic exercise stand out as the most prevalent methods, recognized for their purportedly advantageous effects [14, 15]. There is substantial evidence supporting the recommendation of core muscle exercise therapy to decrease the intensity of back pain and reduce levels of disability in aviation pilots [4, 16] and other populations [17]. Core muscle exercise is specifically designed to enhance the activity of local stabilizing muscles, including the transversus abdominis and lumbar multifidus, representing a significant distinction from traditional exercise modalities [17, 18]. Given the increased demands placed on spine stabilization during accelerating flight, pilots exposed to elevated G-forces but possessing lower strength and trunk muscle resistance are at an increased risk of experiencing LBP [4].

Nevertheless, in clinical practice, electrotherapy represents a noninvasive and nonpharmacological approach to managing back pain, with interferential currents (IFC) [19] being the most frequently employed method. This technique is based on the physiological effects of low-frequency electrical nerve stimulation, delivered without pain or discomfort. One of the primary advantages of IFC lies in its ability to reduce skin impedance, enabling it to penetrate deeper into the tissues. IFC not only enhances circulation and alleviates pain by stimulating large nerve fibers but also restores the neurovegetative balance, thereby modulating the sympathetic nervous system and promoting muscle relaxation, potentially contributing to pain alleviation [20, 21].

Several studies have shown that IFC is effective at reducing pain, and the effects of IFC have largely been explored in patients with painful diseases such as chronic LBP [22, 23], neck pain, soft tissue pain in the shoulder, and myofascial pain syndrome [24]. However, to our knowledge, few studies have analyzed the analgesic effect of a combination of IFC with specific therapeutic exercise in patients with chronic LBP; in particular, there are no relevant studies involving high-performance fighter pilots. While a recent study [22] indicated that the combination of IFC and exercise did not yield any discernible advantages in alleviating disability, it was found that exercise alone could significantly reduce disability among individuals grappling with chronic LBP. Nevertheless, it is worth noting that researchers employing varied therapies in conjunction with exercise did manage to achieve a reduction in pain intensity [22]. Further elucidation of the overall health status of the chronic LBP population undergoing combination therapy remains a topic of ongoing investigation.

Therefore, the present study aimed to investigate the effects of combined IFC with specific core muscle exercises and single therapy methods (IFC or core muscle exercises) on pain, disability, quality of health, and core muscle function in Chinese Air Force Fighter pilots with chronic LBP.

## Methods

### Study design

**Fig. 1:**
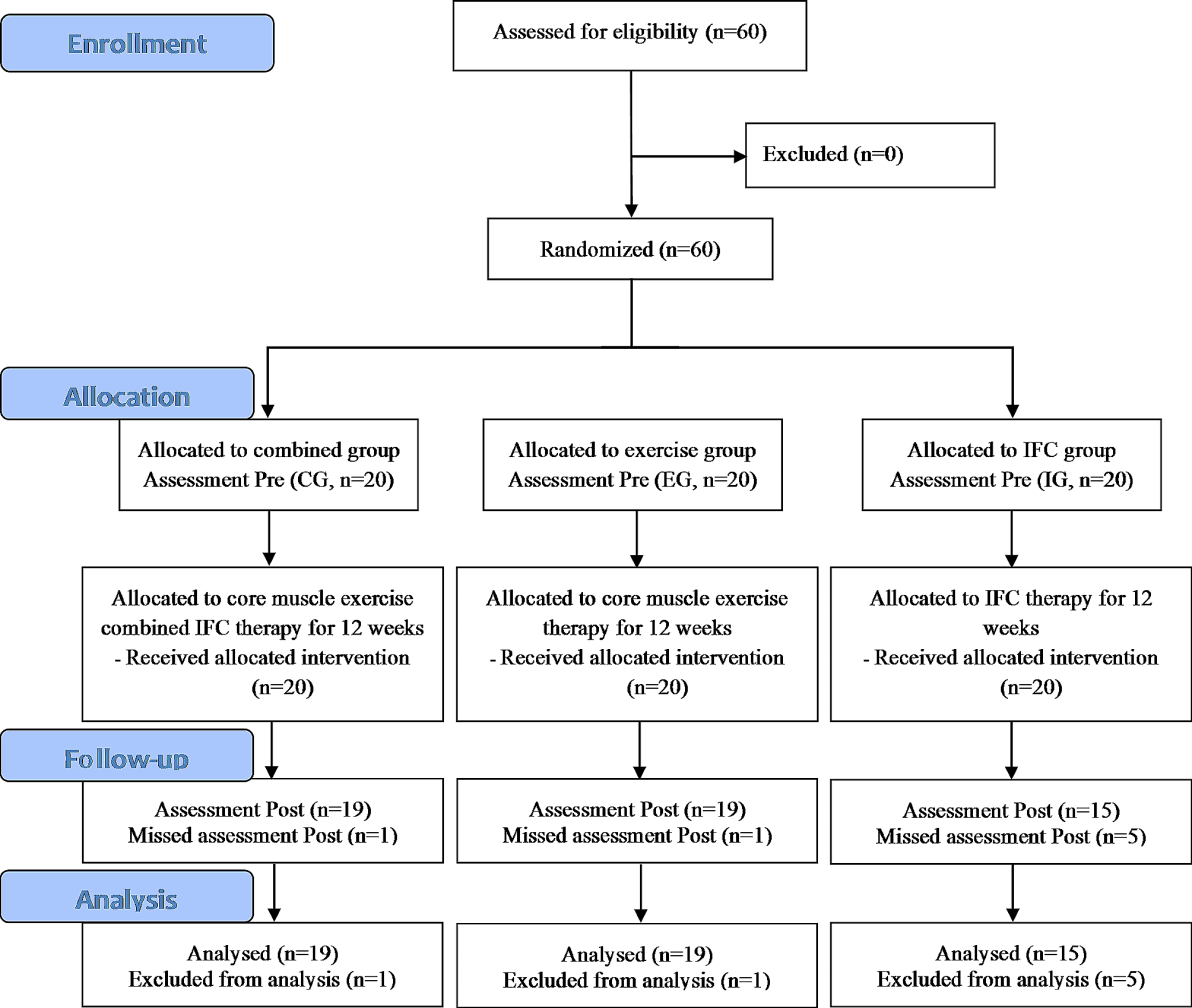
Design and flow of participants through the study following CONSORT 2010 guidelines

A prospective randomized controlled trial was conducted with a blinded assessor. The participants were recruited from military fighter pilots of the Chinese Air Force Base. The current study was approved by the Ethics Committee of the Air Force Medicine Center of the Chinese PLA (protocol number: ChiCTR2300074642). The relative design, conduct and data analysis of this research followed the recommendations of the CONSORT guidelines (Fig. 1). The research team was composed of one principal investigator, one autonomous researcher, and three intervention physiotherapists. The primary investigator furnished all participants with details regarding the study’s protocol and the safety of the associated experiments and treatment methodology, securing informed written consent for participation. Additionally, participants were obligated to fill out a comprehensive medical history questionnaire, which received approval from the Ethics Committee of the Air Force Medicine Center and adhered to the ethical principles outlined in the Declaration of Helsinki.

### Participants

The participants in this study were fighter pilots who presented with chronic non-specific LBP, diagnosed by a rehabilitation physician. Chronic LBP was defined as persistent pain and discomfort localized below the costal margin for a duration of at least 12 weeks or longer. Consequently, a total of 60 male participants, aged between 28 and 56, were ultimately enrolled in the study.

The inclusion criteria in this study were as follows: (1) had chronic LBP for ≥ 3 months or more; (2) were male adults aged between 18 and 56 years (including active duty pilots and flight administrators); (3) were not afraid of electrotherapy; and (4) were willing to join in this study. The exclusion criteria were as follows: (1) had been restricted from flying in the last 3 months; (2) reported pain intensity of less than 2 scores on the VAS in the last 3 months; (4) had any uncontrolled prolapse, intervertebral disc, bone disorders or arthritis; (5) were engaged in core strengthening exercises or under any physiotherapeutic treatment; and (6) were unable to follow routine exercise or not perform timely evaluation procedures.

### Procedure

Following the initial baseline assessment, all participants were randomly assigned to the IFC group (IG, *n* = 20) to receive IFC therapy; the core muscle exercise group (EG, *n* = 20) to receive core exercise for trunk muscle; and the core muscle exercise combined with IFC group (CG, *n* = 20) to receive IFC therapy plus core muscle exercise. The intervention therapy within each group was conducted under the vigilant supervision of a physiotherapist possessing relevant experience working in the hospital. Before randomization, participants were evaluated with several assessments. The primary outcome measures included pain intensity (assessed via the visual analog scale - VAS), the Oswestry disability index (ODI), and quality of life scores (evaluated using the SF-12) to measure their health status. Secondary outcome measures focused on muscle function, including trunk muscle strength (evaluated through the partial curl-up and trunk flexor test), endurance (measured via the Sorensen test), and trunk range of motion (ROM), as well as ROM of hip medial/lateral rotation. All assessments were performed by the same physiotherapist both at the outset and after 3 months of intervention therapy. To ensure consistent load conditions during flight training, variations in flight time and intensity over the course of the year were taken into consideration, and all participants were assessed over a week-long period.

### Randomization and blinding

The randomization procedure was executed through a computerized random number generator using the Research Randomizer Program online (https://www.randomizer.org/). This process allocated each participant to one of three groups, namely, the IFC group, the exercise group, and the combined group, maintaining a balanced 1:1:1 ratio. All participants consented to their respective group assignments, and an independent researcher, uninvolved in the recruitment or intervention phase, conducted this allocation. Furthermore, the randomization schedule was exclusively accessible to the intervention physiotherapist responsible for overseeing the participants.

### Interventions

All participants were allocated into an IFC group, an exercise group or a combined group to receive intervention therapy 5 days per week for 12 consecutive weeks. The patients in each group underwent approximately 30 min (IFC) or 45 min (core muscle exercise) of therapy, which was monitored by an experienced physical therapist.

The protocol for IFC therapy entailed a comprehensive explanation, instructions, and precautionary measures for the participants, who maintained a comfortable prone position with their heads supported by a pillow. The physiotherapist began by sanitizing the painful site using alcohol swabs. Subsequently, four self-adhesive surface electrodes were affixed to the areas of discomfort, connected to an IFC device (longest LGT2800V2, China). The stimulation parameters involved the use of four rubber electrodes, each measuring 5 × 5 cm, with attached sponges. These sponges were moistened with water, and a fixed-frequency current of 4000 Hz, along with a modulated frequency of 4150 Hz, was applied, resulting in an effective frequency of 150 Hz and a current intensity ranging from 20 to 70 mA. The therapist systematically adjusted the IFC intensity based on the participants’ feedback, aiming to achieve a “pins-and-needles” sensation without visible muscle twitches. Each session was conducted over a duration of 30 min.

The core muscle exercise protocol was a specifically progressive program under physiotherapist supervision that was adapted for each participant’s perceived pain and load tolerance and included warm-up (5 min), core stability and strength exercises (30 min), and cool-down (10 min). Following the methods employed in a previous study [25], static stabilization exercises comprised the following: gluteal bridge, side bridge, prone bridge, supine extension bridge, straight leg rise from prone, and alternate arm and leg raise from quadruped. Each exercise was held for a duration of 8–10 s and repeated 10 times, with brief intermissions between exercises. The training intensity progressively escalated with reduced assistance and enhanced performance from each participant. During the isometric contraction phase of these exercises, participants were instructed to engage their abdominal muscles while maintaining a regular breathing rhythm. In contrast to static stability exercises, core strength training adopted a dynamic training approach, as previously recommended. Dynamic core strength exercises specifically targeted the rectus abdominis (RA), abdominus obliquus internus (OI), abdominus obliquus externus (OE), and erector spinae (ES) [26]. The exercises included: For the RA in dorsal decubitus with fixed knees: Trunk flexion to engage the RA muscles. For OI and EO in dorsal decubitus with flexed knees: Trunk flexion and rotation to engage OI and EO muscles. For the RA in dorsal decubitus with semi-flexed knees: Hip flexion to engage the RA muscles. For the ES in ventral decubitus: Trunk extension to engage the ES muscles. Each exercise involved 3–4 sets of 15 repetitions, with a 1-min rest interval between sets. The exercise therapy’s intensity gradually increased over time, with the following progression: for static stabilization exercises, from 8 to 10 s in the initial six weeks to 20–30 s in the subsequent six weeks, repeated 5 times; for dynamic strength exercises, the repetitions increased from 15 to 25.

The protocol for core muscle exercise combined with IFC therapy consisted of the core muscle protocol and the IFC protocol, as mentioned above. First, participants in the CG underwent a 3-day core muscle protocol and 2-day IFC therapy per week during the first six weeks of intervention, and then, they performed a 2-day core muscle protocol and 3 days of IFC therapy per week during the second six-week intervention.

### Outcome measurements

#### Pain intensity and disability

The evaluation of pain intensity and disability was made based on the VAS and ODI scores, respectively. Participants were asked to indicate the subjective level of their pain on a 10-cm horizontal line, ranging from 0 to 10 scores, with circular markers placed before and after the intervention therapy. Zero scores signified the absence of pain, while 10 scores indicated the worst conceivable pain. The VAS has been validated and demonstrates very high test-retest reliability [27]. Participants reported the average intensity of pain experienced during the preceding week of rest. The ODI, on the other hand, is a condition-specific questionnaire that individuals self-administer, known for its validity, reliability, and responsiveness in assessing the extent of disability attributable to chronic LBP. The ODI consists of 10 questions, and each question is described on a 6- score scale ranging from 0 to 5 scores, with a total possible score ranging from 0 to 50 scores. Higher ODI scores indicate more severe disability. The ODI is a valid scale with high test-retest reliability and a Cronbach’s alpha = 0.87 [28].

#### Quality of life

The SF-12 health-related quality of life (QOL) questionnaire, a concise survey, was employed to evaluate the quality of life in participants with chronic LBP. This self-report survey consisted of 12 questions, which was applied to determine health-related QOL directly related to the condition of physical and mental health, and included a physical component summary (SF-12 PSC) and a mental component summary (SF-12 MCS). The SF-12 is considered both valid and reliable, exhibiting moderate test-retest reliability and a Cronbach’s alpha coefficient of 0.85 [29].

### Core muscle function

Core muscle function assessments comprised measuring the maximum isometric strength of the trunk and hip muscles, evaluating trunk muscle endurance, and determining the range of motion of hip medial/lateral rotation. The specific details of each test are presented below.

**Fig. 2:**
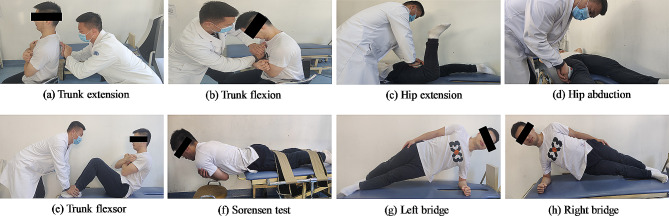
Example core muscle function tests of trunk extension (**a**), trunk flexion (**b**), hip extension (**c**), hip abduction (**d**), trunk flexor (**e**), Sorensen test (**f**), left bridge (**g**), right bridge (**h**)

The strength generated by the trunk and hip muscles during maximum isometric voluntary contraction was assessed using a manual digital dynamometer (MicroFET 2, Hoggan Health Industries, USA). This instrument demonstrated good test-retest reliability in evaluating extension and flexion, as well as rotation strength of the trunk and hip muscles [30, 31]. Initially, participants were familiarized before the measurements were taken. Participants sat on a fixed chair with the backrest and arm crossing the chest while their feet were kept suspended in the air. A manual dynamometer was affixed to the participant trunk by the tester’s two hands as shown in Fig. 2a and b. Subsequently, the dynamometer was positioned at the level of the sternum stem or the height corresponding to the fourth or fifth thoracic vertebra, enabling the participant to resist forward or backward bending. Each test had a duration of 5 s, and the best outcome from three replicates, with a 1-min rest interval between each test, was utilized for the final analysis.

The maximum isometric strength of hip muscle extension and abduction was also assessed using the same dynamometer as shown in Fig. 2c and d. Before the measurements, participants underwent two submaximal trials to familiarize themselves with each test position. This was followed by two maximal isometric contractions for each muscle group, both on the left and right sides. For hip extension testing, participants maintained a prone position on a treatment bed with 0° hip extension and 90° knee flexion. The dynamometer was positioned in the middle of the posterior thigh, allowing the tester to resist extension. In the case of abduction testing, participants were in a supine position with 0° hip extension, and the dynamometer was placed on the lateral supra patella, enabling the tester to resist abduction. Verbal encouragement was provided to all participants during the tests. The final analysis considered the best result from three replicates, with a 30 s rest interval between each trial, and the average of the left and right extensions/abductions was used for further analysis.

Core muscular endurance was assessed without tactile or verbal feedback and was measured in seconds in four positions: trunk flexor, Sorenson (trunk extensor), and side bridge on both sides as shown in Fig. 2e to h. Participants were instructed to maintain each static position for as long as possible. The test sequence was randomized by either a physiotherapist or the main investigator following procedural agreement, including the determination of endpoints. The trunk flexor endurance test was conducted with a 55° angled jig, an arm positioned across the chest, and feet anchored under a foot support. The knees were at a 90° angle, and the upper torso rested toward the jig. The trunk extensor endurance test was performed in the Biering-Sorensen position, with the lower limbs secured and the upper body cantilevered over the edge of the test bench, aligning the anterior superior iliac spine parallel to the bench edge. The test concluded when participants were unable to maintain their current position or exhibited oscillations exceeding 5 cm. Side bridge tests were executed on either side with elbow support, legs fully extended, hips lifted off the ground, and the top foot placed in front of the lower side, creating a straight body alignment. Before starting each test, participants received identical verbal and visual instructions. Additionally, all participants were allowed to rest for 3 min between each test. These test procedures were validated in a previous study [32].

The ROM for hip internal and external rotation was evaluated using a clinical inclinometer (Hoaliangsk, GJJDC01, China). This inclinometer has been validated and shown to be reliable, as described by Van Dillen [33]. Participants were placed in a prone position, with the leg being measured in the neutral hip position at 0° of abduction/adduction and the knee flexed to 90°. The non-test leg was positioned with slight hip abduction. The inclinometer was aligned with the long axis of the distal tibia and adjusted to 0°. Then, the test leg was allowed to rotate internally or externally until the pelvis began to rotate, and the angle was recorded. All measurements of hip rotation ROM were repeated three times, and the most favorable result from these three tests was employed for data analysis.

### Sample sizes

The sample size was calculated using G*power software (3.1.9.4, Düsseldorf University, Germany). To obtain a clinically relevant decrease in pain intensity (approximately 2 scores on the VAS) in chronic LBP patients. Using CG and IG data, we assumed a medium effect size (0.25) and a pooled standard deviation (SD) of 0.9. A fixed medium effect size (0.25) with a power of 80% and a significant alpha (α) level of 0.05 (two-tailed) were used to calculate samples using repeated measures, within-between interactions, analysis of variance, a 3-group design and 2 time points (baseline and after 12 weeks). We estimated the need for 42 total participants in this study. Considering a 10% dropout rate, 47 participants met our recruitment requirements. However, a total of 60 participants provided consent, yielding 20 participants per group.

### Statistical analysis

All statistical analyses were conducted using GraphPad Prism version 8.4 (GraphPad, San Diego, CA, USA). All the data are presented as the means ± SD and were validated to be normally distributed using the Shapiro–Wilk test before being subjected to subsequent statistical analyses. One-way ANOVA was used to examine the between-group differences in baseline and post-intervention demographic variables, health conditions, and core muscle function variables. Two–way repeated measures analysis of variance (time × group) was used to analyze the effects of the three different intervention regimens on the VAS score, ODI, QOL, and muscle function. After ANOVA, Bonferroni post hoc comparisons was used for multiple comparisons. Additionally, Cohen’s d effect sizes were computed as partial eta square (*η*^*2*^), following Cohen’s proposal [34], and categorized as follows: small (< 0.01), moderate (0.01–0.138), or large (> 0.138) for all the data. A statistical significance level of α = 0.05 was applied to all tests.

## Results

Of the 60 participants, 7 withdrew after allocation and introduction to the intervention programs, with 1 dropout from the IG (constraints), 1 dropout from the EG (daily life with time-consuming training sessions), and 5 dropouts from the CG (no desire for further participation). Therefore, the study sample consisted of a total of 53 participants, and the flow chart outlining the participants’ flow throughout the study is illustrated in Fig. 1. Table 1 shows the general characteristics of the fighter pilots included in this study according to group allocation, including anthropometric characteristics; VAS score; ODI score; PCS score; MCS score; years of work; and number of flight hours per week. Furthermore, a one-way ANOVA indicated that there was no significant difference between these groups (*p* > 0.05). All fighter pilots engaged in routine exercise, including resistance training and running, which is a common practice among military personnel. Additionally, no adverse effects related to therapy or exercise were observed among the three groups.

**Table 1:** Comparison of initial anthropometric characteristics, flight hours, year of flight and weekly flight time among the three groups

Initial characteristics	CG(*n* = 19)	EG(*n* = 19)	IG(*n* = 15)	*p*-value
Age (years)	37.5 ± 8.2	40.8 ± 8.1	36.6 ± 7.3	0.264
Weight (kg)	74.5 ± 8.3	75.7 ± 7.6	71.7 ± 6.4	0.315
Height (cm)	175.2 ± 4.5	174.5 ± 4.4	174.2 ± 4.8	0.814
BMI (kg/m^2^)	24.2 ± 2.1	24.9 ± 2.1	23.7 ± 2.6	0.329
VAS	3.9 ± 1.0	3.5 ± 1.3	4.1 ± 0.8	0.250
ODI	12.5 ± 4.2	14.2 ± 4.5	11.8 ± 3.2	0.224
PCS	38.7 ± 5.7	37.8 ± 5.2	36.3 ± 3.2	0.377
MCS	43.5 ± 5.6	41.8 ± 4.3	42.9 ± 5.8	0.587
Flight hours	2061.1 ± 1637.1	2511.1 ± 1493.9	1816.0 ± 1389.1	0.401
Year of flight (years)	13.3 ± 7.9	16.3 ± 8.7	12.8 ± 8.3	0.402
Flight time/week (hours)	4.4 ± 3.7	4.6 ± 1.9	4.8 ± 3.1	0.915

CG, core muscle exercise combined IFC group; EG, core muscle exercise group; IG, IFC group; BMI, body mass index; VAS, visual analog scale; ODI, Oswestry disability index; PCS, physical component summary; MCS, mental component summary

**Fig. 3:**
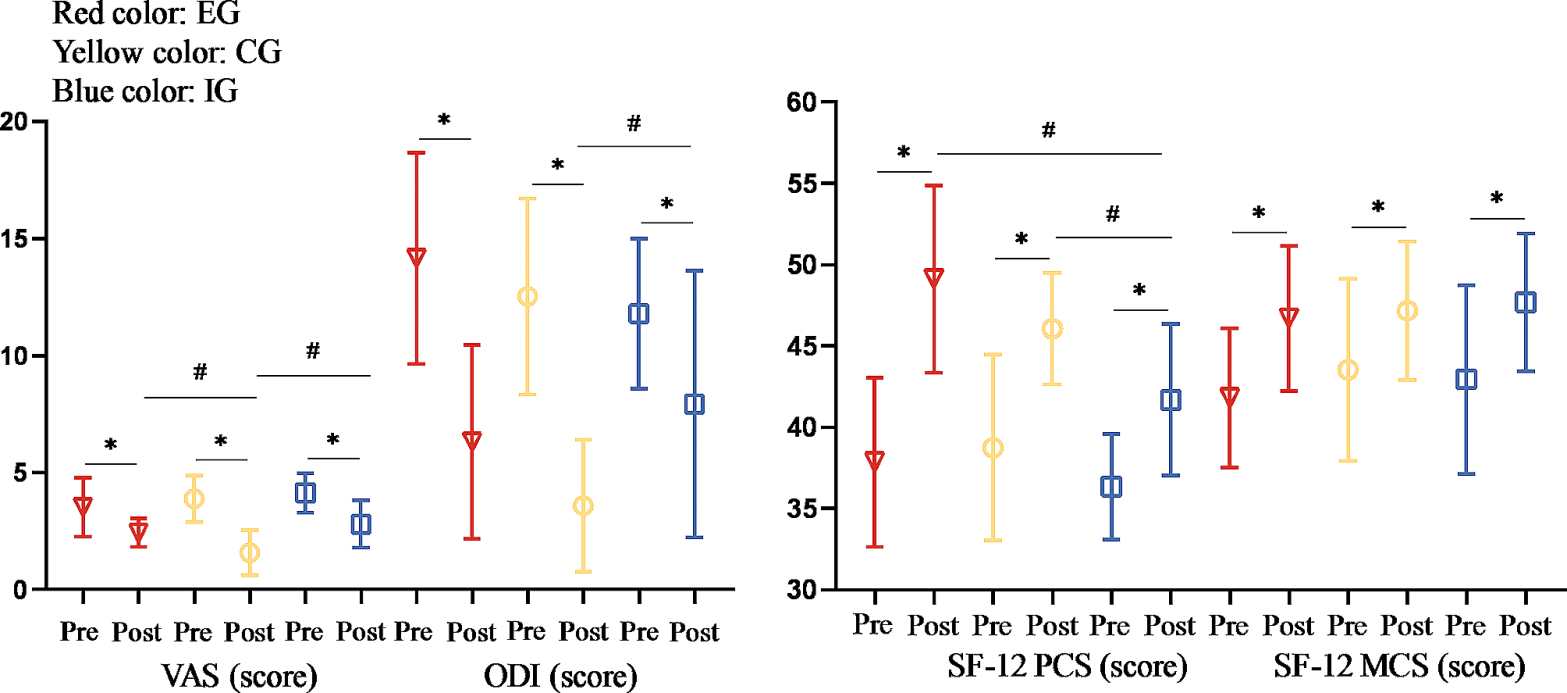
Comparison of scores for self-reported pain intensity, Oswestry disability index, and SF-12 among three groups. EG, core muscle exercise group; IG, IFC group; CG, core muscle exercise combined IFC group; PCS, physical component summary; MCS, mental component summary; *, *p* < 0.05 within group; #, *p* < 0.05 between groups

There was a significant effect of time × group interaction on the VAS score (*p* < 0.01, *η*^*2*^ = 0.213), ODI (*p* < 0.05, *η*^*2*^ = 0.145), and SF-12 PCS score (*p* < 0.05, *η*^*2*^ = 0.138) (Fig. 3). The VAS decreased from pre-to-post-therapy in the EG (Pre = 3.5 ± 1.3 vs. Post = 2.4 ± 1.6, *p* < 0.05), CG (Pre = 3.9 ± 1.0 vs. Post = 1.6 ± 1.0, *p* < 0.05), and IG (Pre = 4.1 ± 0.8 vs. Post = 2.8 ± 1.0, *p* < 0.05), the ODI decreased from pre-to post-therapy in the EG (Pre = 14.2 ± 4.5 vs. Post = 6.3 ± 4.1, *p* < 0.05), CG (Pre = 12.5 ± 4.2 vs. Post = 3.6 ± 2.8, *p* < 0.05), and IG (Pre = 11.8 ± 3.2 vs. Post = 7.9 ± 5.7, *p* < 0.05), and the SF-12 PCS increased form pre-to post-therapy (Pre = 37.8 ± 5.2 vs. Post = 49.1 ± 5.7, *p* < 0.05)in the EG, CG (Pre = 38.7 ± 5.4 vs. Post = 46.1 ± 3.4, *p* < 0.05), and IG (Pre = 36.3 ± 3.2 vs. Post = 41.7 ± 4.7, *p* < 0.05). Post hoc analyses revealed that all groups showed post-therapy improvements in pain intensity, ODI and SF-12 PCS (*p* < 0.01), whereas the VAS improvements were greater in the CG than in the EG [MD = − 0.84 scores, 95% CI, (-1.54 to − 0.15), *p* = 0.013] and in the IG [MD = -1.22 scores, 95% CI, (-1.96 to − 0.48), *p* = 0.000] and ODI [MD = − 4.35 scores, 95% CI, (-7.99 to − 0.72), *p* = 0.014] and in the SF-12 PCS [MD = 4.39 scores, 95% CI, (0.36 to 8.41), *p* = 0.029] at the end of the intervention were greater in the CG than in the IG.

A significant effect of time × group interactions was detected for trunk extension strength (*p* < 0.01, *η*^*2*^ = 0.252) and flexion (*p* < 0.01, *η*^*2*^ = 0.242); trunk flexor muscle endurance (*p* < 0.05, *η*^*2*^ = 0.157); the Sorensen test (*p* < 0.05, *η*^*2*^ = 0.127); the left side-bridge (*p* < 0.01, *η*^*2*^ = 0.175); and the right side-bridge (*p* < 0.05, *η*^*2*^ = 0.157) (see Table 2), but not for hip muscle extension strength (*p* > 0.05, *η*^*2*^ = 0.031) or abduction strength (*p* > 0.05, *η*^*2*^ = 0.038). Bonferroni post hoc comparison analyses in Table 2 revealed that the improvements in maximum isometric strength of the trunk, endurance of trunk flexor, Sorensen test and both sides bridge were significantly greater in the EG and CG than that in the IG (*p* < 0.05).

**Table 2:** One-way ANOVA and Bonferroni correct post-hoc comparison results of core muscle strength and endurance outcomes in different group

Group Variables	One-way ANOVA		EG - CG	EG - IG	CG - IG
	F	p-value			
Trunk extension(kg)	8.546	0.001			
MD			-3.3	6.3	9.7
*p-*value			0.411	0.029	0.000
Trunk flexion(kg)	5.694	0.006			
MD			-1.8	6.3	8.2
*p-*value			1.000	0.044	0.006
Hip extension(kg)	0.219	0.804			
MD			-0.5	0.5	1.0
*p-*value			1.000	1.000	1.000
Hip abduction(kg)	2.262	0.115			
MD			0.1	1.8	1.6
p			1.000	0.170	0.237
Trunk flexor(s)	4.196	0.021			
MD			-0.8	17.6	18.4
*p-*value			1.000	0.047	0.036
Sorensen test(s)	5.981	0.005			
MD			-0.4	18.7	19.1
*p-*value			1.000	0.012	0.010
Left side-bridge(s)	5.383	0.008			
MD			-4.1	15.6	19.7
*p-*value			1.000	0.046	0.008
Right side-bridge(s)	5.407	0.007			
MD			-4.4	15.6	20.0
*p-*value			1.000	0.049	0.008

EG, core muscle exercise group; IG, IFC group; CG, core muscle exercise combined with IFC group; MD, mean difference

Also, we found that only time main effect was observed for the ROM of left hip internal (*p* < 0.01, *η*^*2*^ = 0.238), left external (*p* < 0.01, *η*^*2*^ = 0.162), right hip internal (*p* < 0.01, *η*^*2*^ = 0.318), and right external (*p* < 0.01, *η*^*2*^ = 0.282). No group main effect and time × group interactions were observed for all variables (*p* > 0.05). Bonferroni post hoc comparison analyses in Table 3 revealed no significant difference between EG and CG, EG and IG, or CG and IG (all *p* > 0.05).

**Table 3:** One-way ANOVA and Bonferroni correct post-hoc comparison results of hip internal and external ROM outcome

Group Variables	One-way ANOVA		EG - CG	EG - IG	CG - IG
	F	p-value			
ROM of left hip internal	2.579	0.086			
MD			-4.7	-2.7	2.1
*p-*value			0.083	0.724	1.000
ROM of left hip external	0.090	0.914			
MD			0.7	0.6	-0.1
*p-*value			1.000	1.000	1.000
ROM of right hip internal	2.914	0.064			
MD			-4.2	-1.4	2.8
*p-*value			0.064	1.000	0.427
ROM of right hip external	2.390	0.102			
MD			-3.4	-0.5	2.9
*p-*value			0.136	1.000	0.328

EG, core muscle exercise group; IG, IFC group; CG, core muscle exercise combined with IFC group; ROM, range of motion; MD, mean difference

## Discussion

To the best of our knowledge, the present study represents the inaugural attempt to assess the impact of a specific core muscle exercise combined with interferential current therapy on chronic LBP among high-performance fighter pilots. Administration of core muscle exercise in conjunction with IFC therapy, as well as core muscle exercise or IFC therapy as a standalone treatment, resulted in a significant reduction in the intensity of LBP, a decrease in disability, and an enhancement in quality of life. Nevertheless, the combined therapy exhibited superiority in reducing pain intensity in comparison to the other two therapeutic methods. Moreover, in both the combined therapy group and the solitary core muscle exercise group, notable enhancements were observed in trunk and hip strength, core muscular endurance, and hip rotation range of motion compared to the IFC therapy group. Our findings indicate that high-performance pilots grappling with chronic LBP attain more pronounced improvements in pain reduction, disability alleviation, and enhancement in core muscle function when subjected to either combined therapy or therapeutic exercise, subsequently leading to an augmented quality of life.

The 95% CI indicated a significantly greater reduction in pain intensity and disability following the 12-week therapy intervention for participants who received combined therapy compared to those who received either single therapeutic exercise or IFC therapy. Specifically, concerning pain intensity, this randomized trial revealed a more substantial improvement in the combined therapy group, with a decrease in clinical pain by 2.3 points on a 10-point scale, surpassing the practical minimum worthwhile effect threshold of 2.1 points [35]. Notably, only the combined therapy approach achieved this desirable threshold. Fortunately, all three groups experienced a significant reduction in pain intensity after therapy, consistent with earlier studies demonstrating that exercise therapy, IFC therapy, or combined therapy for chronic LBP leads to a substantial decrease in pain intensity [18, 22, 36]. However, this study is the first to establish that the combination of core muscle exercise and IFC therapy was more effective in reducing pain and disability among fighter pilots with chronic LBP.

Chronic LBP is associated with the strength and endurance of trunk muscles, and an imbalance in hip muscles can contribute to the occurrence of LBP. Fighter pilots with chronic LBP experience greater pain intensity when they are in a longer sitting position. The prolonged maintenance of a static posture can lead to fatigue during the eccentric contraction of back muscles and may serve as a significant factor contributing to flight-related pain experienced by military aviators operating different types of aircraft [37]. The advantage of core muscle exercises is that LBP weakens abdominal and lower back muscles [38], leading to a significant reduction in pain intensity in the combined group. Several previous studies involving helicopter pilots have suggested that incorporating core strengthening exercises and stabilization exercises into a treatment plan could enhance the strength of weakened muscles, alleviate tension, reduce anterior pelvic tilt, and ultimately alleviate spinal pressure, resulting in pain relief [16, 36, 39]. These findings align with the outcomes of our study on core muscle exercises. Similar to our present study, prior research has indicated that there is no discernible difference between IFC and other methods, such as muscle release techniques [40], manipulation [41], or motor improvement exercises [42], for both acute and chronic LBP. However, the integration of IFC therapy with core muscle exercises produced superior results in terms of reducing pain intensity. This can be attributed to the additional effects of IFC stimulation, which generates amplitude-modulated frequency parameters, eliciting low-frequency currents deep within the treatment area by interacting with two medium-frequency circuits. By modulating the frequency’s amplitude, it becomes possible to stimulate nerves and other tissues, including muscles, ligaments, and lumbar joints. This stimulation can control pain by activating the pain-gating mechanism and triggering descending pain suppression mechanisms [43]. Therefore, the findings of our study may hold clinical significance for rehabilitation and healthcare teams involved in the treatment of chronic LBP in fighter pilots.

Patients with chronic LBP who achieve an improvement of at least 6 points in the ODI score are typically categorized as experiencing a “moderate” improvement, which is generally considered a worthwhile effect [44]. Regarding disability, as measured by ODI, Ostelo and de Vet [45] proposed that a minimum clinically important difference should involve a 10-point threshold change following an intervention. However, caution is advised when applying this threshold, as it may not always adequately identify meaningful clinical changes in certain studies. In our present study, improvements in ODI appeared to result in a worthwhile effect for the participants. We observed a significant reduction in ODI compared to baseline after the 12-week combined therapy in the CG (ODI difference of 7.9 scores), following core muscle exercise in the EG (ODI difference of 8.9 scores), and with IFC therapy in the IG (ODI difference of 3.9 scores). These findings align with previous studies that have demonstrated a significant reduction in ODI when using either core exercise or IFC therapy alone, both in helicopter pilots [4, 36] and the general population [46]. Although the findings of comparable effects of combined or single therapy on pain and disability in patients with chronic LBP observed in this study are consistent with the findings of several previous studies, these findings still differ from the conflicting reports of other studies on the superior efficacy of combined therapy and therapeutic exercise [22]. Before intervention therapy, participants in each group had lower QOL scores than those in the general population [47]. After 12 weeks of therapy, each group showed significant improvement in PCS and MCS scores despite not matching the reference values. Ulger et al. [48] also reported significant improvements in QOL (SF-PCS and SF-MCS) after 18 sessions of core stability exercise and manual therapy over 6 weeks in chronic LBP patients. These results suggest that treatment involving core muscle exercises indeed enhances trunk strength, improves spinal stability, and reduces stress on the spine in CG and EG participants and is effective at ameliorating disability and QOL in daily life activities. Considering that the high incidence of incapacity and disqualification for flying are associated with older age and health conditions, as well as the relatively low average age and high enthusiasm for rehabilitation [49], all participants were strongly able to maintain their health; these factors may be the main reasons for the rapid recovery of QOL in fighter pilots.

Young pilots are considered an important backup resource for fighter pilots, and early initiation of specific treatment is a valid strategy for reducing and preventing musculoskeletal complaints in young fighter pilots to avoid deterioration over the years. In this study, IFC therapy alone did not improve core muscle function parameters, whereas it did improve core muscle function parameters in the core muscle exercise group and combined therapy group. Considering the crucial role of core muscles in chronic LBP, it has been emphasized that exercises aimed at enhancing muscle strength and endurance are essential [50]. Numerous studies have proposed that stabilization exercises effectively enhance core stability and function among individuals with chronic LBP. However, these studies often assessed core stability and function through measures such as core muscular endurance or lumbopelvic functional tests. For instance, Javadian et al. [51] reported that core stability exercises in combination with routine exercise are more effective in improving pain intensity, disability, and core muscular endurance when compared to routine exercise alone in patients with lumbar segmental instability. Shamsi et al. [52] reported that both core stability and traditional trunk exercise decrease pain and disability and improve lumbopelvic function, but no significant difference was observed between the two exercise methods in patients with chronic LBP.

Notably, the present study adopted a specific core muscle exercise, including core stabilization exercises and core strength exercises, which is the greatest difference from the above research programs, but equal improvements could be obtained in terms of core muscle function parameters. Furthermore, incorporating IFC therapy into core muscle exercise represents the initial endeavor in addressing chronic LBP among fighter pilots, with core muscle strength and ROM assessments included in the evaluation of core muscle function. Additionally, it has been documented that the multifaceted aspects of core muscle function include not only endurance and functionality but also variables like strength, flexibility, and ROM [53]. In the literature on evidence-based medicine, clinical trials have directed their attention to diverse facets of core muscle function, and these trials are widely acknowledged for their high reliability [53]. In this study, we used core strength, endurance, and ROM tests to assess core muscle function and found that combined therapy and core muscle exercise were superior to IFC therapy alone for improving core muscle function parameters, namely, strength and endurance, which could be related to improvements in core motor control. Previous studies have shown that better trunk endurance and strength are related to better postural stability [54, 55]. Although the use of IFC alone resulted in slight improvements in core muscle function parameters following 12 weeks of intervention therapy, these changes were not statistically significant.

Several prior studies have investigated the influence of various exercise modalities on core muscle function by assessing the contractility and myoelectric activity of associated muscles [4, 38]. Nevertheless, these techniques, including ultrasound scanning and electromyography, furnish objective data. Nevertheless, in specialized research settings and among specific populations, a straightforward, cost-effective, easily executable, and dependable evaluation holds importance. Consequently, the assessment of core muscle function, as carried out in our study and the parameters that we have previously established as highly reliable, can be executed by clinicians and researchers to appraise the effects of therapeutic intervention on all facets of core function.

This study has several potential limitations. The main limitation of the current study is the absence of follow-up data, and the effect of therapeutic intervention was assessed after 12 weeks of intervention. Future studies are necessary to determine whether the effects of each therapeutic method on pain intensity, disability, QOL, and core muscle function are longer. Second, we experienced a relatively high drop-out rate in the IFC group, which left us with a relatively low number of participants who had completed IFC therapy alone, and the lack of samples may have led to bias in the experimental results. Third, due to the lack of objective assessment equipment, such as ultrasound scanning and electromyography equipment, it is impossible to determine the actual changes in the muscles. However, further studies will be needed in the future to overcome these limitations.

## Conclusion

In conclusion, the outcomes of this study illustrate that the advantages derived from the combination of core muscle exercise with IFC are noteworthy. Core muscle exercise and IFC therapy in isolation appear effective in reducing pain, diminishing disability, and enhancing quality of life. However, the combined therapy stands out by significantly alleviating pain and reducing disability compared to the individual therapies. Furthermore, both the combined therapy and core muscle exercise offer comparable advantages concerning core muscle function. In light of these findings, we propose the adoption of the combined therapy approach as the primary treatment option for fighter pilots afflicted with chronic LBP in clinical practice, aiming to enhance their health condition. Moreover, we recommend conducting further investigations to ascertain the duration of these benefits.

## Data Availability

All data generated and analyzed during this study are included in this published article. The raw data supporting the conclusions of this article will be made available by the authors, without undue reservation, to any qualified researcher. First author Chongwen Zuo should be contacted if someone wants to request the data in reasonable.

## Abbreviations

ARR: Absolute risk ratio
CG: Core muscle exercise combined IFC group
EG: Core muscle exercise group
IG: IFC group
IFC: Interferential current
LBP: Low back pain
NNT: Number needed to treat
ODI: Oswestry disability index
QOL: Quality of life
ROM: Range of motion
RR: Risk ratio
RRR: Relative risk ratio
SF-12 PCS: Short Fomr-12 physical component summary
SF-12 MCS: Short Fomr-12 mental component summary
VAS: Visual analogue scale
